# Online National Health Agency Mask Guidance for the Public in Light of COVID-19: Content Analysis

**DOI:** 10.2196/19501

**Published:** 2020-05-26

**Authors:** Linnea Laestadius, Yang Wang, Ziyad Ben Taleb, Mohammad Ebrahimi Kalan, Young Cho, Jennifer Manganello

**Affiliations:** 1 Zilber School of Public Health University of Wisconsin Milwaukee Milwaukee, WI United States; 2 College of Nursing and Health Innovation University of Texas Arlington, TX United States; 3 Robert Stempel College of Public Health Florida International University University Park, FL United States; 4 School of Public Health University at Albany State University of New York Albany, NY United States

**Keywords:** public health policy, infectious disease, personal protective equipment, public health, COVID-19, pandemic, online health information, content analysis

## Abstract

**Background:**

The rapid global spread of the coronavirus disease (COVID-19) has compelled national governments to issue guidance on the use of face masks for members of the general public. To date, no work has assessed how this guidance differs across governments.

**Objective:**

This study seeks to contribute to a rational and consistent global response to infectious disease by determining how guidelines differ across nations and regions.

**Methods:**

A content analysis of health agency mask guidelines on agency websites was performed in late March 2020 among 25 countries and regions with large numbers of COVID-19 cases. Countries and regions were assigned across the coding team by language proficiency, with Google Translate used as needed. When available, both the original and English language version of guidance were reviewed.

**Results:**

All examined countries and regions had some form of guidance online, although detail and clarity differed. Although 9 countries and regions recommended surgical, medical, or unspecified masks in public and poorly ventilated places, 16 recommended against people wearing masks in public. There were 2 countries that explicitly recommended against fabric masks. In addition, 12 failed to outline the minimum basic World Health Organization guidance for masks.

**Conclusions:**

Online guidelines for face mask use to prevent COVID-19 in the general public are currently inconsistent across nations and regions, and have been changing often. Efforts to create greater standardization and clarity should be explored in light of the status of COVID-19 as a global pandemic.

## Introduction

The rapid global spread of the coronavirus disease (COVID-19) has compelled national governments to issue guidance on the use of face masks for members of the public. Growing evidence of transmission from asymptomatic and presymptomatic individuals makes the development of these guidelines increasingly pressing [1-3]. Recent research suggests that surgical masks could help prevent transmission of human coronaviruses by reducing emissions of coronavirus RNA in respiratory droplets and aerosols [4]. Although N95 respirators have the potential for even greater protection when compared to surgical masks [5,6], they also require fit testing that make them unsuitable for the public at large [7]. Currently, both surgical masks and N95 respirators are in short supply, with health care workers continuing to face shortages of personal protective equipment (PPE). In light of this, fabric masks have become a third masking option, although current evidence on their efficacy is limited. Prior studies suggest that fabric masks are significantly less effective than surgical masks, both for protecting health care workers and for reducing spread among the general public [8,9]. Even with lower efficacy, however, all masking options appear to hold value. Recent modeling suggests that widespread public adoption of even relatively ineffective masks would be able to help curtail community transmission of COVID-19, although more effective masks yield greater reductions in mortality [10].

Despite growing evidence on the value of masking and calls for public use of masks as part of a broader strategy that also includes social distancing and hand washing [10,11], recent commentary suggests that public guidance on masks may be inconsistent across nations [12], and the World Health Organization (WHO) maintains, as of May 2020, that masks are only needed for healthy individuals when they are taking care of someone with suspected COVID-19 [13]. Given the pandemic status of COVID-19, it is critical to establish a baseline understanding of current government guidelines on mask use for the general public. To date, no studies have conducted a systematic analysis of mask guidelines aimed at the public. Public-facing guidelines are critical to compliance since government provision of cues is an important driver of mask use [14]. Agency websites are a particularly critical way to disseminate these types of guidelines, as the public increasingly turns to the internet for health information.

To inform this discussion and help health agencies to “adopt rational recommendations on appropriate face mask use” [12], this paper presents a content analysis of health agency mask guidelines in March 2020 among countries and regions with large numbers of COVID-19 cases.

## Methods

The 25 countries and regions with the highest number of confirmed COVID-19 cases were drawn from the Johns Hopkins Center for Systems Science and Engineering Coronavirus COVID-19 Global Cases tracker on March 9, 2020 [15]. These countries and regions are listed in Table 1. To replicate the experience of someone looking for guidance, we visited national health agency websites for each country and region seeking mask guidelines (medical, surgical, and unspecified mask types) aimed at the public. Given limited evidence for their efficacy relative to surgical and medical masks [8,9], we considered fabric masks separately. Specifically, we sought to find both recommendations for or against fabric masks for primary use and recommendations for fabric masks only when other more effective masks are unavailable. A content analysis approach was used [16], and a codebook was developed in Excel (Microsoft Corporation) to track guidance on when masks are recommended or not recommended. Initial coding suggested that several nations indicated that masks were not recommended because they may increase risks or create a false sense of safety. A code was also added to track these statements. Countries were assigned across the coding team by language proficiency, with Google Translate used as needed. When available, both the original and English language version of guidance were reviewed. All relevant webpages and documents were downloaded to create a static record. Websites were coded between March 13 and March 23, 2020, with all coding verified by a second coder the following week. Any coding discrepancies were discussed among authors and resolved. All websites were revisited a final time on March 30 to look for updated materials, and coding was updated as needed.

**Table 1:** Health agency guidance for public use of surgical, medical, and unspecified masks for coronavirus disease as of March 30, 2020.

Country/region	Public should wear masks when symptomatic	Public should wear masks when caring for/in proximity of symptomatic people	Public should wear masks when in public places/places with poor ventilation	Masks are explicitly not recommended for the public at large	Masks may pose health risks or create a false sense of security
Australia	✓^a^	X^b^	X	✓	X
Austria	✓	✓	✓	X	X
Bahrain	✓^c^	X	X	X	X
Belgium	✓	X^d^	X	✓	X
Canada	✓	✓	X	✓	✓
France	✓	X	X	✓	X
Germany	✓	X	X	✓	✓
Greece	✓	✓	X	✓	X
Hong Kong	✓	✓	✓	X	X
Iran	✓	✓	✓	✓^e^	✓^e^
Iraq	X	X	✓^f^	X	X
Italy	✓	✓	X	✓	✓
Japan	✓	✓	✓	X	X
Kuwait	✓	✓	✓	X	X
Mainland China	✓	✓	✓	X	X
Malaysia	✓	✓	✓	X	X
Netherlands	X	X	X	✓	✓
Norway	✓	✓	X	✓	✓
Singapore	✓	X	X	✓	X
South Korea	✓^g^	✓^h^	✓	X	X
Spain	✓	X	X	✓	X
Sweden	X	X	X	✓	X
Switzerland	✓	X	X	✓	✓
United Kingdom	X	X	X	✓	X
United States	✓	✓	X^i^	✓	X

^a^✓: guidelines were identified on the website.

^b^X: guidelines were absent on the website.

^c^Bahrain requires masks when in self-isolation for 14 days following a return from a country with a high volume of coronavirus disease cases.

^d^Belgium indicates that “wearing face masks to prevent coronavirus infection only makes sense in hospitals where patients with Coronavirus are treated.”

^e^Iran’s newer guidelines recommend masks, but the old document discouraging public mask use still remains active on the health agency website.

^f^Iraq lacks formal guidelines but featured a press release about the importance of wearing masks when shopping.

^g^South Korea recommends a KF94 or higher respirator rather than a surgical or unspecified medical mask when caring for coronavirus disease cases.

^h^South Korea recommends a KF80 or higher respirator when symptomatic.

^i^The United States recommends the use of fabric masks in public places as of April 3, 2020, but explicitly does not recommend surgical mask use.

## Results

All 25 countries and regions had some form of publicly available information about masks on their health agency websites aimed at the public. Format and level of detail ranged greatly and included infographics (eg, Malaysia) and short responses in a frequently asked questions format (eg, Netherlands). Iraq had the vaguest guidance, which the coding team inferred from news stories and press releases about mask use rather than the existence of a formal page or document with public COVID-19 prevention guidance. A total of 4 (16%) countries and regions lacked recommendations for wearing surgical, medical, or unspecified masks when symptomatic, and 12 (48%) countries did not mention use by individuals providing care during home quarantine (Table 1). Although 9 (36%) countries and regions recommended surgical, medical, or unspecified masks in public or poorly ventilated places, 16 (64%) explicitly recommended against the general public wearing masks. A total of 7 (28%) also noted that surgical, medical, or unspecified masks were not recommended because they could increase health risks to the wearer or give a false sense of security.

With regard to fabric masks, no countries or regions recommended this mask type as preferable to surgical or medical masks in the Table 1 scenarios. Out of the 25 countries and regions, there was a country and a region (n=2, 8%) that explicitly recommended against them due to their protective capacity being either unknown (Italy) or inadequate (Hong Kong). South Korea and Mainland China recommended fabric masks as part of a broader guidance of different mask types being appropriate for different risk scenarios. South Korea, for example, noted: “In cases where there is not a high risk of infection or there is no health mask, it is helpful to use a cotton mask (including replacing the electrostatic filter) to avoid droplets directly from coughing or sneezing.” Germany, the United States, and Japan recommended some form of fabric mouth covering (including scarves and handkerchiefs) only when other options were unavailable. Austria was the only country or region at the time of analysis to recommend fabric masks interchangeably with other types of masks, noting that a “textile mouth-nose guard can also be used” as part of guidance on mask use in public spaces. The remaining 17 (68%) countries and regions did not explicitly address fabric masks in their guidance.

In some cases, countries or regions updated their guidance during the study period. For example, Iranian guidance initially recommended asymptomatic individuals not wear masks. By March 29, 2020, additional guidance was posted recommending masks at the park, gym, or when engaging in urban travel. On March 30, Austria removed guidelines referencing the WHO that stated that “disposable face masks are not an effective protection” and instead recommended “protective mask[s] in public spaces where there may be close contact with other people, e.g. in supermarkets.” By contrast, Sweden scaled back guidance during the study period, removing language that masks could help prevent spread from symptomatic individuals.

## Discussion

### Principal Findings

As of late March 2020, there was little consistency in guidance on face mask use for the public, despite COVID-19 being declared a global pandemic. Although the countries and regions analyzed were chosen in light of having the highest number of confirmed cases in early March 2020, per-capita rates varied considerably. Accordingly, some of the variation in guidance could be due to countries or regions being in different stages of pandemic response. Guidance may also be informed by strategic considerations related to PPE shortages and a desire to reserve masks for health care providers. However, variation in statements regarding mask risks suggest a more fundamental difference in assessments of masks as an appropriate approach for reducing community spread of COVID-19. Many differences also appear to be regional. With the exception of Austria, only Asian and Middle Eastern nations and regions recommend masks of any type in public as of March 30, 2020. This is also broadly consistent with greater mask use in Asian nations during previous outbreaks such as H1N1 and severe acute respiratory syndrome [17,18]. Several European countries also failed to outline guidelines consistent with the WHO recommendation that symptomatic individuals and those who care for them should wear masks.

The United States in particular has struggled with face mask guidelines. In early March 2020, the US Surgeon General issued a strongly worded tweet indicating that members of the public should not purchase masks in response to the spread of COVID-19, suggesting both that masks would be ineffective and that they are needed by health care providers [19]. The US Centers for Disease Control and Prevention (CDC) also consistently advised the public not to use face masks unless sick or caring for someone sick and denied that any updated mask guidance was scheduled as of March 28, 2020 [20]. On April 3, 2020, the CDC updated its website guidance to recommend that the public wear fabric masks in public settings where social distancing is a challenge [21]. The following day the US Surgeon General posted a video on Twitter demonstrating how to make a face mask out of a T-shirt [22]. Guidelines also specify that they are not recommending surgical masks, as these “are critical supplies that must continue to be reserved for health care workers and other medical first responders” [21]. At the time of writing, US states continue to face shortages of PPE [23], and the CDC recommends medical use of bandanas and scarves as a last resort [24]. The United States should monitor the efficacy of its guidelines relative to those in other nations and regions.

As illustrated by the US example, guidelines are constantly evolving in light of new risk information and mask availability. Although the ability to shift guidelines is critical to ensure that they reflect current evidence, changes also pose distinct health communication challenges. For example, some members of the public may struggle to understand why universal mask use is encouraged if the previous message focused on masks posing a health risk. Misinformation about mask use already appears to be circulating on social media [25]. Research on understanding and receptivity to mask guidance will be critical. Most recently, several countries in addition to the United States appear to be rethinking the value of fabric masks. For example, both Iran and Greece now provide online instructions for how to create a fabric mask at home [26,27]. It will remain important to maintain awareness of developments in mask guidelines across regions and nations given that COVID-19 is not bound by political and legal borders. It is also imperative that mask guidelines are clearly communicated to the public with messages explaining any guideline changes.

### Limitations

Findings on mask guidance should be interpreted in the context of their limitations and recognition that the sample focused on countries and regions with high levels of COVID-19 in early March 2020. Although we sought to assign coding based on language proficiency, some countries and regions necessitated more reliance on Google Translate than others. This may have introduced some translation errors. Some nations and regions may have communicated information via social media that was not present on their website and, therefore, not included in this analysis. Finally, mask guidance does not necessarily imply mask access, and the availability of masks for public use is a separate question that warrants significant attention from researchers and policy makers.

### Conclusions

Although COVID-19 was declared a pandemic by the WHO on March 11, 2020 [28], guidelines for face mask use to prevent COVID-19 in the public remained broadly inconsistent across nations and regions at the end of March 2020. Efforts should be made to continue to monitor mask recommendations and create greater standardization based on scientific evidence. Furthermore, there is a strong need for additional research on the efficacy of different mask types in community settings. Although not the primary focus of this study, the clarity of guidelines was also a source of concern, with some guidelines spread across multiple pages and sometimes not specifying the type of mask recommended. Further, as mask use begins to increase in nations and regions where face masks have not experienced “cultural assimilation”[18], it will be critical to expand guidelines to include not just *when* masks should be worn but also *how* they should be worn. Future research should consider how to best communicate such guidelines to the public, particularly as guidelines continue to change over time.

## Abbreviations

CDC: Centers for Disease Control and Prevention
COVID-19: coronavirus disease
PPE: personal protective equipment
WHO: World Health Organization
